# The effect of a short, animated story-based video on COVID-19 vaccine hesitancy: A study protocol for an online randomized controlled trial

**DOI:** 10.3389/fpubh.2022.939227

**Published:** 2022-08-23

**Authors:** Sandra Barteit, Violetta Hachaturyan, Ferdinand Beleites, Tilman Kühn, Caterina Favaretti, Maya Adam, Till Bärnighausen

**Affiliations:** ^1^Heidelberg Institute of Global Health (HIGH), Faculty of Medicine and University Hospital, Heidelberg University, Heidelberg, Germany; ^2^Institute for Global Food Security (IGFS), Queen's University Belfast, Belfast, United Kingdom; ^3^Professorship of Behavioral Science for Disease Prevention and Health Care, Technical University of Munich, Munich, Germany; ^4^Department of Pediatrics, Stanford University School of Medicine, Stanford, CA, United States; ^5^Africa Health Research Institute (AHRI), Durban, South Africa; ^6^Harvard Center for Population and Development Studies, Cambridge, MA, United States

**Keywords:** COVID-19, vaccine hesitancy, short video, animated videos, public health, randomized control trial (RCT)

## Abstract

**Introduction:**

Exposure to a high volume of vaccine misinformation on social media can have a negative effect on vaccine confidence and rates. To counteract misinformation, we designed a collage of three short, animated story-based (SAS) videos to convey scientifically informed and accessible information about COVID-19 vaccine applicable to a social media context.

**Methods and analysis:**

We will conduct an online randomized controlled trial primarily to: (1) determine the effectiveness of SAS videos in improving COVID-19 vaccine knowledge; (2) evaluate the effectiveness of SAS videos in increasing behavioral intent for COVID-19 vaccination; and (3) quantify people's interest in watching SAS videos about the COVID-19 vaccine. We also aim to identify barriers and facilitators to COIVD-19 vaccinations that have been shown to minimize vaccine hesitancy between vaccinated and unvaccinated populations. Using a web-based recruitment platform, a total of 10,000 adults from the United States will be recruited and randomly assigned to (1) a SAS video collage arm, (2) an attention placebo control video arm, or (3) no intervention arm (1:1:1). Furthermore, we will measure behavioral intent to obtain information on vaccination regarding COVID-19. At the end of the trial, participants randomized to arm 2 and arm 3 will be given the option of watching one of the intervention videos voluntarily to assess participant engagement with SAS videos. Finally, we will assess individual factors associated with vaccine hesitancy - hope, optimism, COVID-19 perceived risks and benefits, self-efficacy, perceived social norms, and trust - and compare vaccinated and unvaccinated participants across the three arms.

**Discussions:**

Evidence-based information from official channels can be complex and inaccessible to the general public, whereas false information on social media is frequently shared in brief postings, images, or videos that can easily reach the general public, thereby rapidly disseminating (mis-)information. To avoid the spread of misinformation, social media may be used to deliver evidence-based and emotionally compelling information in a readily accessible format in order to pre-empt misinformation. Our findings may help inform future SAS efforts addressing COVID-19 and other important public health challenges.

**Ethics and dissemination:**

The study was approved by the Heidelberg University Hospital's Ethics Committee (S-163/2022). The trial was registered with German Clinical Trials Register (www.drks.de) on 5 January 2022: number DRKS00027938. Findings of the study will be published in peer-reviewed scientific publications and possibly presented at scientific conferences.

## Introduction

Vaccination programs are key components in preventing and decreasing the prevalence of numerous vaccine-preventable diseases, and have historically had a considerable impact on public health (1–3). Yet, the COVID-19 pandemic has seen an upsurge in vaccine hesitancy described as a “delay in acceptance or refusal of vaccination despite availability of vaccination services” (1). For individuals, vaccine hesitancy substantially increases the risk for more severe COVID-19 infections. At the same time, a higher number of severe, clinical COVID-19 cases constitutes a significant challenge for healthcare systems (2). Hesitation and lack of trust in vaccines are longstanding problems that impeded prevention efforts during earlier pandemics, including outbreaks of the Severe Acute Respiratory Syndrome (SARS), Ebola Virus Disease, and Middle East Respiratory Syndrome (MERS) (3). A complex range of social and individual factors have been shown to impact vaccine hesitancy, including public health policies, education and income levels, risk perceptions, trust in authorities, and, importantly, misinformation (4). Since the outbreak of the COVID-19 pandemic, false information and speculative reports about COVID-19 vaccines began circulating on social media platforms, threatening to undermine public trust and confidence in vaccines (5, 6). While vaccination rates have increased globally, anti-vaccination efforts and conspiracy theories continue to spread on social media, eroding public confidence in vaccination. Several studies have shown that vaccine misinformation is associated with lower vaccination rates and higher vaccine resistance (7, 8). By the end of 2021, 10.9% of U.S. adults over 18 years old were still hesitant about getting vaccinated against COVID-19 (9). Recent data also reveals that misinformation and hesitancy is spread even among fully vaccinated individuals. Not all people eligible for a COVID-19 booster dose are willing to get it (10). A fully vaccinated individual may accept or reject a booster dose for a variety of reasons, including vaccination-related adverse effects, perceived effectiveness of the booster dose, susceptibility to the respective infection, and safety concerns. Therefore, measures to increase COVID-19 vaccination and counter disinformation cannot be limited to individuals who have never received a COVID-19 vaccination. Numerous studies have reported that misinformation spreads substantially faster, broader, and wider on social media than factual information (11–13). One reason may be that online information provided by official channels is overly complex and inaccessible in comparison to social media posts, which frequently feature visually appealing images or short videos. As this format is more accessible to the general population, it seems to foster the rapid spread of (mis-)information. Existing literature on health communication indicates that using entertainment-education, particularly animated educational videos, is a successful strategy for developing persuasive, evidence-based health messages and promoting health literacy (14–16). According to research, including relevant narratives into health messages may be more effective than presenting health messages as factual arguments alone (17). Social media platforms have the potential to rapidly disseminate short evidence-based educational content, making reliable information about COVID-19 vaccinations more accessible to the general population. Short, animated story-based (SAS) videos, in particular, may be useful as a novel kind of health message approach since they combine instructional content with an entertainment component to match popular content types on social media platforms (18). Research findings suggest that SAS videos are a preferred format among participants for receiving health messages on a wide range of public health topics (18, 19). Furthermore, SAS videos were shown to have a positive impact on health-related knowledge and behavior (19–21).

As part of this study, we prepared a collage of three SAS videos about the COVID-19 vaccine with no audio (just sound effects and background music) that illustrate the importance of getting vaccinated against COVID-19. The employed SAS videos incorporate a variety of audience engagement methods, including culturally agnostic characters and an emotionally compelling soundtrack, that may encourage more users to share the videos on social media (22). We will evaluate the effectiveness of the SAS videos in increasing knowledge about the COVID-19 vaccination, as well as the impact of the SAS videos in changing attitudes and behavioral intent to get vaccinated and learn more about the COVID-19 vaccine. Additionally, we will examine the degree of participants' voluntary involvement, as defined by their willingness to view the SAS videos and the duration of time spent completing the task in the non-intervention arms. Lastly, we will examine the differences in individual characteristics related with vaccine confidence between the three arms - such as hope, optimism, risk and benefit perception, self-efficacy, perceived social norms, and trust. Our findings will support others in developing simple yet effective health communication strategies to address vaccination hesitancy more effectively.

The primary objectives of this study are as follows:

To evaluate the SAS video's effectiveness in:

- improving COVID-19 vaccine knowledge.
- increasing behavioral intent toward COVID-19 vaccine.
- quantifying intrinsic interest in watching a SAS video about COVID-19 vaccine.

The secondary objective of this study is to explore the differences in individual factors connected to vaccine hesitancy between the three study arms after the interventions, which are equally distributed across groups by randomization.

## Methods and analysis

### Trial design

**Figure 1:**
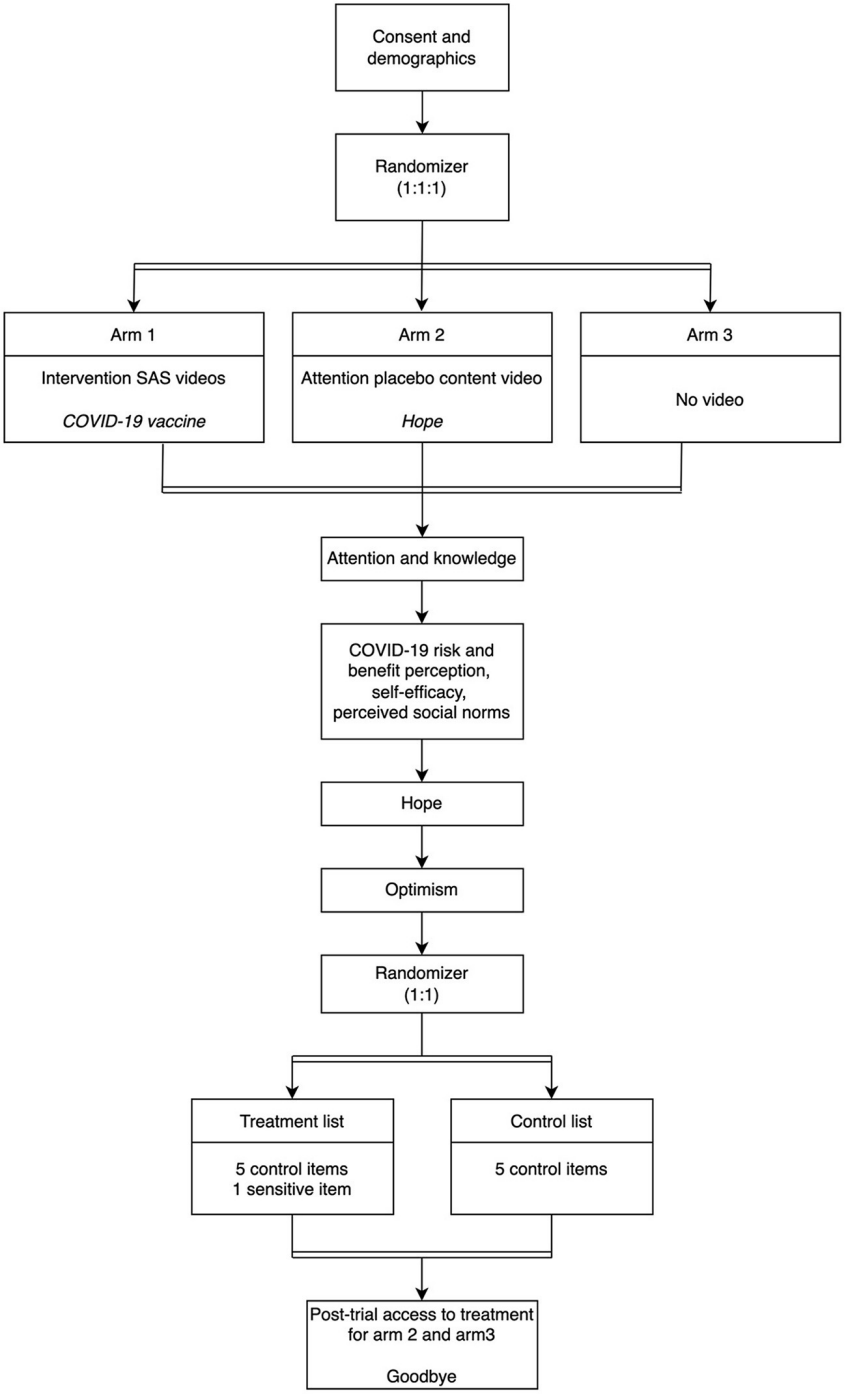
Design of randomized controlled trials (second randomization is for a list experiment rather than for the group assignment). The order of the attention, knowledge, hope, and optimism questionnaires is sequential, since study participants complete them in succession. Inclusion criteria: Participants must be over the age of 18 (male, female, or other), have a current address in the United States, and be proficient in English.

We will conduct a randomized controlled trial (RCT) following a multi-site, parallel group design with post-trial access to treatment (see Figure 1). After completing demographic, vaccine, and political beliefs surveys, participants will be randomly assigned to one of three arms: an intervention arm exposing participants to the SAS video collage (arm 1), an attention placebo control (APC) arm showing a video of similar length unrelated to the vaccine (arm 2), or a control arm without any intervention (arm 3). We will randomize the participants in a 1:1:1 ratio to the trial arms. In the intervention arm, participants will watch a collage of three SAS videos underlining the importance of COVID-19 vaccination to provide study participants with access to evidence-based information regarding their risk of contracting and spreading the COVID-19 virus (arm 1: COVID-19 vaccine message) (23). In the APC arm, participants will watch a video that shows how important it is to help children find hope in the future (arm 2: no COVID-19 vaccine message) (24). In the control arm, participants will receive no intervention (arm 3: no video). Participants in arm 1 and arm 2 will not be able to pause or skip the video.

After watching the video, participants will be asked to complete one attention question, four true/false knowledge questions about the COVID-19 vaccine, 14 Likert scaled questions about COVID-19 vaccine perceived risks and benefits, attitude, self-efficacy, and perceived norms, and 11 additional Likert scaled items indicating their level of hope and optimism. All questionnaires incorporating Likert scaled items were obtained from our prior research and are cited in the following sections that discuss the questionnaire items.

Furthermore, participants in each arm will be asked to complete five list experiments (25). For each list experiment, we randomized participants 1:1 to a control list or a treatment list. The control list consists of a list of four items about behavioral intent, which are unrelated to the COVID-19 vaccine. The treatment list consists of the same four list items as the control group, as well as a sensitive item assessing behavioral intent to get vaccinated and obtain additional information about COVID-19 vaccinations. The list experiment will be employed to decrease social desirability bias, as participants may already be primed to respond positively to questions about COVID-19 vaccines. Following completion of the list experiment, study participants will respond to seven questions regarding their level of trust in governmental bodies, institutions, and health care providers.

Study participants randomized to the APC group (arm 2) or the control group (arm 3) will be given the option to watch the COVID-19 vaccination intervention video as a post-trial access to treatment at the end of the experiment. The participants will be informed that they will not receive compensation for the time spent watching the SAS videos.

Participants are expected to complete the experiment in 10 min or less. If for whatever reason, participants take longer than 45 min to finish the survey, they will be timed out of the online experiment platform and will not be able to take the survey again. The time-out period is intended to prevent participants from clogging the system with incomplete surveys. We are unable to initiate follow-up within the 45-min time restriction due to the participants' confidentiality.

A pilot phase will evaluate the trial setup as described here within a smaller sample size and shorter study duration, to ensure that the main trial proceeds as seamless as possible.

### Study setting

The proposed RCT will be conducted online. The experiment will be designed and implemented using Gorilla (Cauldron Science Limited) (26), a platform for designing and conducting online behavioral experiments. Participants may participate in the study using a mobile phone, tablet or computer.

### Participants

Participants must be over the age of 18 (male, female, or other), have a current address in the United States, and be proficient in English. Participants who do not meet the inclusion criteria will be excluded from the study. We will recruit study participants stratified by age and gender in order to replicate the age-sex distribution of the adult population in the United States, and vaccination status (no doses, one, two, three or more doses received; or tested COVID-19 positive).

### Recruitment

Participants will be recruited using Prolific (Prolific Academic Ltd) (27), an online, academic research platform for recruiting participants for research studies. Participants must create an account on Prolific and provide their personal information. Each user is assigned a unique, anonymized ID. Every 48 h Prolific sends an email to a random subset of all eligible users registered on the platform. Participants are enrolled on a “first come, first served” basis until the recruiting limit is met. Participants who do not match the eligibility conditions are automatically excluded by Prolific. Participants in the study will be paid £1.70 for completing the survey. Prolific will be handling reimbursement of research participants. Participants are not permitted to take the survey twice with the same ID, per the platform's restrictions. Prolific controls the accounts, hence it is impossible for us to determine if a user has many accounts. We are unaware of the platform's methods for preventing such incidents.

#### Blinding

Due to the fact that recruiting will take place on the Prolific platform, study participants will be unable to be identified or data linked back to them. Participants will respond to the survey questions and submit their responses anonymously through the Gorilla platform. Both, study participants and investigators of this study, will be blinded to the allocation status of the participants.

#### Concealment mechanism

The Gorilla platform will complete the randomization by using a web-based randomization algorithm.

#### Sequence generation

Gorilla will randomly allocate participants to each of the study arms. Gorilla allows for two randomization options: (1) independent randomization of each individual based on a probability drawn, and (2) balanced randomization, which randomizes without replacement so that a fixed number of study participants end up in each study arm. To ensure that our experimental arms are balanced, we will employ the “balanced randomization” option.

### Sample size calculation

We are interested in determining if there is a difference in knowledge across three different groups (arm 1, arm 2, arm 3). We used a one-way analysis of variance to determine the sample size for pairwise comparisons across the three groups with the software R (package pwr.anova.test). We assumed the alpha level to be 0.05 and power to be = 0.80, and an effect size of *f* = 0.04. This calculation led to about *n* = 1,300 study participants per group, that is a total of 3,900 study participants. As per a prior study conducted (18), for the control and treatment groups, we assumed a mean of μA = 2.0 and μB = 2.15, respectively (in other words, we expect, on average, that the control group will agree with 2 out of the 5 items and the treatment group will agree with 2.15% of the 6 items). We selected σA = 0.85 and σ = 1.0; this calculation yields a sample size of *n* = 769 per group. For a 5-way comparison, the sample size is *n* = 3,845.

We will choose a sample size of *n* = 10,000 to guarantee appropriate power and to account for attrition. No mathematical correction will be made for multiple comparisons.

For the pilot study, we deemed a sample population of 10% of the full sample sufficient (*n* = 1,000).

### Intervention

The intervention consists of a collage of three SAS videos educating viewers about the importance of getting the COVID-19 vaccine to stop the spread of the virus. Created by our co-author MA for Stanford Medicine, all videos are animated with sound effects but do not include any words, speech, or text. The total duration of the collage is 4 min. The first video tells the story of a grandmother and her three family members, who are absorbed in their digital devices and completely ignore their grandmother. When a healthcare professional knocks on the door and offers them the COVID-19 vaccine, the daughter declines while the grandmother welcomes the healthcare professional and gets vaccinated. Visibly pleased, the grandmother explains to the family that polio and other infectious and deadly diseases would still exist without vaccination. Finally encouraged, the other family members agree to get the COVID-19 vaccine. In the second video, the fight against the coronavirus is represented in a video game scenario. The coronavirus is an evil particle that gains points by infecting people and levels up (develops into a variant) by jumping from person to person. The virus' triumph is over as soon as the people decide to deploy the COVID-19 vaccine as a defense which turns out to prevent new variants from emerging. The main character of the third video is an unhappy and sad man who walks down the street and is denied access to shops, clubs, and means of transportation. Suddenly he meets a doctor who offers him a shot against COVID-19. After getting vaccinated, all those activities forbidden to him up to that moment are now available and his mood changes from forlorn to happy. To maximize cross-cultural appeal, the figures were purposefully depicted without distinguishable cultural identification, while the background music is entertaining and geared to increase interaction and user sharing of the videos on social media (28). The videos are interspersed with black screens reporting short facts about the COVID-19 vaccine.

We will compare the SAS intervention videos about COVID-19 vaccine with an APC video (arm 2), and no video (arm 3). Developed by our co-author (MA) for Stanford Medicine in collaboration with the IASC, UNICEF, and the WHO, the APC video is similar in style to the SAS intervention videos; it is also animated, with a duration of 4 min, and aims to convey messages of hope, solidarity and empowerment to kids and their caregivers (no health or COVID-19 vaccine-related messages). The APC video will mimic the inactive component of the intervention, i.e., the video format, but not the active component of the intervention, i.e., the message about COVID-19 vaccine. This will allow the content effect of the COVID-19 vaccine intervention videos to be isolated.

Lastly, the comparison between the intervention SAS videos against COVID-19 and the no-video task will allow us to measure the total effect of the intervention.

### Outcomes

The primary outcome of this study is the effectiveness of the SAS intervention video in increasing knowledge about the COVID-19 vaccine. While knowledge alone is insufficient to modify health behaviors, it is vital for individuals to access and comprehend their health options in order to exert greater control over their health decisions (29, 30). Furthermore, as a second primary outcome, we will measure changes in behavioral intent toward the COVID-19 vaccination. According to the Theory of Planned Behavior (TPB), the intention to act is considered the immediate determinant of action. In this study, behavioral intent is defined as a participant's commitment to receive the COVID-19 vaccine and to seek information about the vaccine during the next two weeks (31). As a third primary outcome, we will evaluate participant willingness (quantification) to voluntarily engage with the SAS intervention videos (post-trial access to treatment) to determine whether SAS videos may have the potential to generate interest as a precursor for health promotion and communication.

As secondary outcomes, we will focus on COVID-19 risk and benefit perception, social norms, hope, and optimism that we hypothesize will change as a result of the intervention.

#### Primary outcome measures

The specific primary outcomes are to improve COVID-19 vaccine knowledge, to increase behavioral intent toward COVID-19 vaccine, and to quantify intrinsic interest in watching a SAS video about COVID-19 vaccine.

##### Attention questions

We will conduct attention questions to establish whether interventions were actively viewed and not merely skipped or played in the background while the person engaged in other tasks (one information attention question per video). Respectively, the question pool comprises the following questions:

1. The video describes the difference between Pfizer and Moderna vaccines (arm 1) – [False].
2. The video is about a fantasy creature who travels the world helping children find hope in the future and joy in simple pleasures (arm 2) – [True].

##### Knowledge

To determine if the SAS intervention video increases participants' knowledge of the COVID-19 vaccine, all participants will be asked true/false questions that are spread over a total of three parts:

**Part 1**

Which of the following are diseases that have nearly been eradicated by vaccines? Choose True or False for each:

1. Polio has nearly been eradicated by vaccines.
2. Smallpox has nearly been eradicated by vaccines.
3. Malaria has nearly been eradicated by vaccines.
4. Rubella has nearly been eradicated by vaccines.
5. Tuberculosis has nearly been eradicated by vaccines.
6. Lyme Disease has nearly been eradicated by vaccines.
7. Measles has nearly been eradicated by vaccines.
8. HIV has nearly been eradicated by vaccines.
9. Mumps has nearly been eradicated by vaccines.
10. Tetanus has nearly been eradicated by vaccines.
11. West Nile Fever has nearly been eradicated by vaccines.
12. Diptheria has nearly been eradicated by vaccines.

**Part 2**

1. It is not recommended for pregnant and breastfeeding women to get vaccinated against COVID-19.
2. It is not recommended for immunocompromised people to get vaccinated against COVID-19.

**Part 3**

Which of the following are common side effects of vaccination with the COVID-19 vaccine?

1. Sore arm is a common side effects of vaccination with the COVID-19 vaccine.
2. Chest pain is a common side effects of vaccination with the COVID-19 vaccine.
3. Fever is a common side effects of vaccination with the COVID-19 vaccine.
4. Chills are a common side effects of vaccination with the COVID-19 vaccine.
5. Nausea is a common side effects of vaccination with the COVID-19 vaccine.
6. Shortness of breath is a common side effects of vaccination with the COVID-19.
7. Racing heart is a common side effects of vaccination with the COVID-19 vaccine.
8. Body aches are a common side effects of vaccination with the COVID-19 vaccine.
9. Headache is a common side effects of vaccination with the COVID-19 vaccine.
10. Body rash is a common side effects of vaccination with the COVID-19 vaccine.

##### COVID-19 perception

In addition, we ask about the participants' current attitude regarding COVID-19 and their self-perceived risk assessment:

1. I believe COVID-19 is severe.
2. I believe COVID-19 is serious.
3. I believe COVID-19 is significant.
4. I am at risk for COVID-19.
5. It is likely that I will get COVID-19.
6. It is possible that I will get COVID-19.

##### Behavioral intent

A list experiment approach will be used to assess behavioral intent to be vaccinated as well as obtain and disseminate credible and evidence-based information on the COVID-19 vaccine (see Table 1 for list experiment and respective list items). The control group will be given a list of four items, while the treatment group will be given the same list plus one additional sensitive item. The sensitive item comprises the willingness to read information about the COVID-19 vaccine from official sources, speaking with a health professional about a COVID-19 vaccine, receiving the required dose of a COVID-19 vaccine, learning about the risks and benefits of receiving a COVID-19 vaccine, and sharing information about the COVID-19 vaccine. Participants will be asked how many of the items on the list they agreed with, without indicating which ones. The minimum score for each list is zero, and the maximum score for the control list is four, and five for the treatment list. We designed the list items to minimize ceiling and floor effects (32). We will assess the proportion of participants who agreed with the sensitive item, that is, behavioral intent toward SAS created by health professionals, by comparing the average difference between the treatment and control lists. The items in each list will be randomly arranged to prevent exposing the participant to the purpose of the list experiment, as well as to eliminate order effects (random order).

**Table 1:** List experiment items (treatment item is *underlined*).

**Lists**	***List items***
**List 1: Education**	*In the next 2 weeks, I will…* • Go grocery shopping for me/my family. • Read the latest news on social media channels. • Pick a fight with my partner about COVID-19 precautions. • Use an online grocery delivery service. • *Read information from official sources, like about COVID-19 vaccine*.
**List 2: Healthy behavior**	*In the next 2 weeks, I will..*. • Wash fruits and vegetables with soap. • Do the laundry. • Learn more about alternative medicine. • Wash my hands every day for at least 20 s. • *Talk to a health worker about COVID-19 vaccine*.
**List 3: Moral obligation**	*In the next 2 weeks, I will..*. • Go out with my friends on at least one evening. • Visit a local community health center. • Develop my own smartphone app for tracking the spread of COVID-19. • Take out the garbage at least once per month. • *Get the needed dose of COVID-19 vaccine*.
**List 4: Perceived action efficacy (benefits)**	*In the next 2 weeks, I will..*. • Write and publish my own article about COVID-19. • Spend time chatting with my friends online. • Practice meditation daily. • Do the vacuuming in my house/apartment. • *Learn about risks and benefits of getting a COVID-19 vaccine*.
**List 5: Advocacy**	*In the next 2 weeks, I will..*. • Clean kitchen counters and dishes after use. • Have alcoholic drinks on at least three evenings. • Spend time on the internet. • Measure my body temperature at home daily. • *Share information about COVID-19 vaccine from official sources with my friends and family*.

Each list experiment will be preceded by the question “How many of the five/six statements do you agree with? We don't want to know which ones, just answer how many. In the next 2 weeks, I will...”

##### Participant engagement

At the end of the study, we will offer participants randomized to arm 2 and arm 3 the option to watch the intervention video (post-trial access to treatment) or end the study. The Gorilla platform will record this response and the time spent watching the video. The participants will be informed that they would not be compensated for the additional time taken to watch the intervention video.

#### Secondary outcome measures

Components of the complexity of vaccine hesitancy that have been identified in the research, namely COVID-19 risk and benefit perception, social norms, hope, and optimism, are secondary outcomes that we hypothesize will change as a result of the intervention. Therefore, all participants will be asked to complete questionnaires measuring their perceptions of severity and susceptibility, response efficacy, attitude, self-efficacy, social norms, as well as questions relating to optimism, hope, and trust.

##### Perceived severity and susceptibility

Perceived severity and susceptibility to COVID-19 vaccine will be assessed with three items adapted from Witte et al. (33) and Nabi and Myrick (34), each with three items on a 7-point Likert scale anchored by strongly disagree (1) and strongly agree (7). The three items for severity will be:

1. I believe that COVID-19 vaccine is severe.
2. I believe that COVID-19 vaccine is serious.
3. I believe that COVID-19 vaccine is significant.

The three susceptibility items will be as follows:

1. I am at risk for COVID-19.
2. It is likely that I will get COVID-19.
3. It is possible that I will get COVID-19.

##### Perceived response efficacy

Three items adapted from Witte et al. (33) and Nabi and Myrick (34) will assess perceived response efficacy. Possible responses will be arranged along a 5-point Likert scale, ranging from 1 (strongly disagree) to 5 (strongly agree).

1. Getting vaccinated against COVID-19 works in preventing COVID-19 disease.
2. Getting vaccinated against COVID-19 is effective in preventing COVID-19 disease.
3. If I get vaccinated against COVID-19, I am less likely to suffer from COVID-19 disease.

##### Attitude

Attitude toward COVID-19 vaccine will be assessed by asking participants to rate their agreement, from 1 (strongly disagree) to 7 (strongly agree), with two statements adapted from Nabi and Myrick (34):

1. I feel that getting vaccinated against COVID-19 is a wise thing to do.
2. I think that getting vaccinated against COVID-19 is more trouble than it is worth (reverse-coded).

##### Perceived self-efficacy

To measure self-efficacy, which refers to a person's self-confidence in performing a desired behavior (35), participants will be asked to rate their agreement on a 7-point Likert scale, ranging from 1 (strongly disagree) to 7 (strongly agree). The following three items were adapted from Witte et al. (33) and Nabi and Myrick (34):

1. I am able to get vaccinated against COVID-19 to prevent COVID-19 disease.
2. Getting vaccinated against COVID-19 to prevent COVID-19 disease is easy to do.
3. Getting vaccinated against COVID-19 to prevent COVID-19 disease is convenient.

##### Perceived social norms

Four statements adapted from Quinn et al. (36) will be used to measure social norms, which comprise both perceived and observed regulations, as well as conventions and behaviors of others:

Descriptive norm (1 – few; 5 – nearly all).

1. How many of the people in the US do you think got the COVID-19 vaccine?

Subjective norm (1 – few; 5 – nearly all).

2. Of the people close to you, what proportion wants you to get the COVID-19 vaccine?

Moral norm (1 – not at all; 4 – very strongly).

3. It is my moral obligation to other people to get the COVID-19 vaccine.

Injunctive norm (1 – no expectation; 2 – encouraged; 3 – required).

What is the expectation at your workplace/school when it comes to the COVID-19 vaccine?

##### The adult hope scale

We will use the Adult Hope Scale to evaluate hope. The scale measures a person's level of hope according to Snyder's definition of hope as “a positive motivational state that is based on an interactively derived sense of successful (a) agency (goal-directed energy), and (b) pathways (planning to meet goals)” (37). The scale consists of 12 items, each measured using a 8-point Likert scale (1 = Definitely False; 2 = Mostly False; 3= Somewhat False; 4 = Slightly False; 5 = Slightly True; 6 = Somewhat True; 7 = Mostly True; 8 = Definitely True):

1. I can think of many ways to get out of a jam.
2. I energetically pursue my goals.
3. I feel tired most of the time.
4. There are lots of ways around any problem.
5. I am easily downed in an argument.
6. I can think of many ways to get the things in life that are important to me.
7. I worry about my health.
8. Even when others get discouraged, I know I can find a way to solve the problem.
9. My past experiences have prepared me well for my future.
10. I've been pretty successful in life.
11. I usually find myself worrying about something.
12. I meet the goals that I set for myself.

Of the 12 items, 4 make up the Agency sub-scale (2, 10, 11, 13) and 4 make up the Pathways sub-scale (1, 4, 6, 8). The remaining 4 items are fillers.

##### Optimism

Optimism will be quantified using the validated scale of Brandtstädter and Wentura (38). The following five items will be rated on a 4-point Likert scale (1=strongly disagree, 4=strongly agree):

1. I am looking forward to the life ahead of me.
2. For me the future is full of hope.
3. Thinking about my future makes me worry.
4. I look to the future with confidence.
5. The future holds a lot of good in store for me.

##### Trust

Following Schmelz and Bowles' model (39), participants will be asked to rate their degree of trust in the (i) federal government, (ii) state government, (iii) experts from science, (iv) media, (v) medical professionals, and (vi) their social circle (family, friends, and colleagues) on a scale of 1 to 7 (1 = no confidence at all, 7 = a great deal of confidence).

Additionally, individuals will be asked to rate the degree to which they believe their country's government has been truthful about the coronavirus outbreak (1 = very untruthful, 5 = very truthful).

##### Follow-up with study participants

We will follow up with participants in a range of 4–8 weeks after the intervention to determine retention of COVID-19 vaccine knowledge, behavioral intent toward COVID-19 vaccination, and intrinsic interest in watching and sharing a SAS video about COVID-19 vaccine, by asking study participants:

1. Since your initial participation in this study, have you gotten the COVID-19 vaccine?
2. Have you received another vaccine after your initial participation in this study?
  a. If yes, did the short-animated animation influence your decision to receive a vaccination?
  b. If not, please explain why did not get a vaccination?

3. Have you shared the short-animated video with others?
  a. If yes, with whom?
  b. If no, why not?

4. Have you discussed what you have learned in this study with others?
  a. If yes, what topics of this study did you discuss with others?
  b. If yes, who did you discuss with?
  c. If no, please explain why you did not discuss what you have learned in this study with others?

### Risk management and mitigation

There are no foreseeable risks to participating in the online study as we will not collect any biomarker specimens, provision of interventions or treatments, or clinical recommendations to participants. Participants volunteer and consent to participate in the study and can withdraw at any time. The study will stop once the total estimated sample size of 10,000 study participants is reached; and a total sample size of 1,000 study participants for the pilot study. The participants may not directly benefit from this research. However, we hope that the results of the study will inform future vaccination efforts which may indirectly have broader population benefits.

### Data collection

The Gorilla platform will be used to collect data. By clicking on the response buttons, participants will submit data. We will conduct a pilot phase for the duration of 4 weeks (currently planned for May 2022); the full trial is estimated to last 3 months (currently planned for June to August 2022) or until 10,000 study participants have been recruited, whichever comes first. The overview of the study timeline for our project is depicted in Table 2.

**Table 2:** Overview of study timelines.

**Item**	**Month**											
	**1**	**2**	**3**	**4**	**5**	**6**	**7**	**8**	**9**	**10**	**11**	**12**
Develop protocol												
Obtain ethical clearance												
Register trial with pre-analysis plan												
Publish protocol paper												
Conduct pilot study												
Conduct data analysis pilot study												
Prepare and publish pilot study manuscript												
Revise full study based on pilot study findings												
Conduct full study												
Conduct data analysis pilot study												
Prepare and publish full study manuscript												

### Data management

Each study participant will be allocated a unique, anonymous string identifier. The identifier will be linked to the participant's responses on the Gorilla platform. Participants' unique identifiers will be devoid of any identifying information. The trial data will be stored on Gorilla's cloud platform, which is hosted on Microsoft Azure in the Republic of Ireland. The study investigators will retain ownership of the research data produced. The Gorilla platform enables research investigators to generate and access anonymized data. The data will be downloaded and safely stored in a computing system maintained by the University of Heidelberg. Data will be stored for 10 years (and afterwards deleted) on secured University servers of HIGH, according to good scientific practice.

### Data analysis

#### Statistical methods for primary and secondary outcomes

In a first step, we will describe the sample, including the proportion of respondents by gender, age group, education level, race/ethnic group, vaccination status, and political beliefs. To assess the effectiveness of the SAS intervention video in boosting knowledge about the COVID-19 vaccination, we will first construct a knowledge score by allocating one point for each valid question and zero points for missing or erroneous responses. We will next compute a comprehensive COVID-19 vaccination knowledge score by adding the right answers ranging from 0 to 24. We will use a Kruskal-Wallis test, the model is:


(1) $$\begin{array}{r} KnowledgeScore_{i}=\alpha_{0}+\alpha_{1}VideoArm_{i} \end{array}$$

where *KnowledgeScore*_*i*_ is the total number of knowledge statements correctly answered by participant *i* and *VideoArm*_*i*_ denotes the treatment arm assigned to participant *i*.

We will average the response for the treatment and control lists in each of the three trial arms for each list experiment. We estimate participants' behavioral intent toward COVID-19 vaccination by examining the difference between the treatment and control lists. The content effect will be calculated as the difference in mean scores on a scale of 0–100 between the COVID-19 intervention arm using these estimates. Additionally, we will compute the total intervention impact as the difference in mean COVID-19 vaccination intervention and control arm scores on a scale of 0–100.

These estimates will be obtained by describing the principal and interaction terms in an ordinary least squares (OLS) regression model as follows:


(2) $$\begin{array}{l} \begin{array}{ccc} BehaviouralIntent_{i} & = & \beta_{0}+\beta_{1}VideoArm_{i}+\beta_{2}TreatList_{i} \end{array}\\ \begin{array}{cc} \;+ & \beta_{3}(VideoArm_{i}\times TreatList_{i}) \end{array} \end{array}$$

where *BehaviouralIntent*_*i*_ is the number of statements in the list agreed upon by the participant, *VideoArm*_*i*_ indicates the *k*th arm, and *TreatList*_*i*_ indicates assignment to the treatment or control list. We will calculate standard errors, 95% confidence intervals, and *p*-values for linear combinations of coefficients from the OLS model.

Our third objective is to determine whether study participants in the non-intervention arms are willing to voluntarily engage with the SAS video about the COVID-19 vaccine at the end of the study. The decision to view the SAS video and the duration of time spent watching the SAS video will be used to determine participant engagement. We will create a dummy variable that equals 1 when the participant clicks the “Play” button to view the SAS intervention video and 0 when the participant chooses to skip the SAS intervention video and click “Finish” the study to assess the participant's willingness to watch the SAS intervention video. We will employ a logistic approach to measure the sociodemographic parameters that influence the decision to watch or not watch the intervention video post-trial:


(3) $$\begin{array}{r} WatchVideo_{i}=\gamma_{0}+\gamma_{1}VideoArm_{i}+\gamma_{2}X \end{array}$$

where *y*_*i*_ is a dummy variable which equals 1 when the participant clicks on the “Play” button to begin watching the SAS intervention video and 0 otherwise, *VideoArm*_*i*_ indicates the *k*th arm, *X* is a vector of covariates.

Among the participants who will choose to watch the SAS intervention video, we will quantify the length of time that they will spend watching the SAS intervention video using the timestamps provided by Gorilla's graphical experiment builder. We will use ordinary least squares (OLS) regression models to investigate which sociodemographic factors were associated with engagement time.


(4) $$\begin{array}{r} EngagementTime_{i}={\text{λ}}_{0}+{\text{λ}}_{1}VideoArm_{i}+{\text{λ}}_{2}X \end{array}$$

where *EngagementTime*_*i*_ is the length of time participant *i* spent watching the SAS intervention video reported in seconds (min = 0, max = 1,800), *VideoArm*_*i*_ indicates the *k*th arm, *X* is a vector of covariates.

Regarding our second objective, we will explore the differences in individual factors related to the levels of vaccine hesitancy between the vaccinated and unvaccinated study participants. To do so, we will first compute a comprehensive score for each individual factor which are: perception of risk and benefit, attitude, self-efficacy, perceived social norms, and trust. We will then inspect correlations between such factors and participants' vaccination status using OLS models.

#### Methods in analysis to handle protocol nonadherence and any statistical methods to handle missing data

Participants who do not complete the survey will be subjected to an intention-to-treat (ITT) analysis; ITT may more accurately reflect the real world, given it is likely that individuals do watch online content.

## Discussion

### Principal findings

Promoting COVID-19 vaccine uptake necessitates a deeper understanding of the numerous underlying factors that differentiate vaccine hesitant individuals from responsive individuals. One key component of a communication strategy is to increase access to evidence-based information tailored to individual behaviors and concerns (40). Prior studies have shown high potential of entertainment-focused educational media as a powerful tool for promoting healthy behaviors (41–43). However, only a few studies have explored the potential of SAS videos in tackling vaccine hesitancy (44, 45). In a systematic review, Shen and Han (46) concluded that techniques for measuring the impact of entertainment-focused instructional media are lacking, and they advocated conducting controlled studies to determine what features may result in desirable effects. Given its broad and diverse user base, social media may be an effective platform for conveying and disseminating educational content regarding the benefits of COVID-19 vaccines, which may translate into vaccine uptake. The use of SAS videos, which have previously been shown to be effective in influencing various health behaviors (47–52), may be a promising digital health strategy to engage audiences in evidence-based health promotion leveraging the reach of various social media platforms. The overall objective of this study is to examine the effectiveness of SAS videos in disseminating evidence-based information on COVID-19 vaccination in terms of increasing behavioral intent toward vaccination. Additionally, we will examine SAS videos' capacity to engage a diverse audience. Furthermore, we will examine the differences between vaccinated and unvaccinated populations in terms of individual characteristics associated with vaccine hesitancy. Our findings may contribute to the expanding body of entertainment-focused educational videos by guiding the design and development of future SAS videos with targeted health messages for public health promotion.

### Strengths and limitations

This study will use an RCT design to assign individuals to watch a collage of three SAS videos about COVID-19 vaccine (arm 1), an APC video (arm 2), or no video (arm 3). The videos in arm 1 and arm 2 are identical in length (~4 min each) and similar in style. The randomization to the three trial arms will reduce the likelihood that systematic differences in groups may influence outcomes and will let us isolate the true effect of the SAS intervention videos.

The inclusion of the APC video is a novel component of our study that will allow us to quantify the content effect of the intervention. We define the difference between the SAS intervention videos and the control as the total effect, the difference between the SAS intervention videos and the APC video as the content effect, and the difference between the APC video and the control as the attention effect. There have been several entertainment-education studies that have employed an experimental approach to assess the effect of an intervention video in this way (47–51), but only few on COVID-19 (44, 45, 52).

In each of the three arms, we will run a list experiment to measure behavioral intent while eliminating social desirability bias. It is likely that participants will already be primed to give socially acceptable responses to questions about their views on the COVID-19 vaccine. The indirect questions (i.e., how many statements do you agree with) provide protection to participants if they want to reject the vaccine message without revealing this intention. To the best of our knowledge, few studies have employed a list experiment approach to assess the efficacy of an entertainment-education video in improving a specified health outcome (51, 52). Hence, we are aware that during the study our strategy could prove to have unforeseen disadvantages. Moreover, list experiments are focused on short-term effects of the intervention, which leaves us with uncertainty regarding the question if a SAS video would actually lead participants to get vaccinated in the near future.

Another objective is assessing participants' voluntary engagement with the SAS videos among participants assigned to the APC and control arms (post-trial access to treatment). The findings of this study will assist us in evaluating participants' willingness to watch online SAS videos, particularly when this willingness is weighed against a time cost, simulating a real-world scenario.

Due to the online nature of our study, we may expect to receive responses from participants who happen to be online at the time the study is launched or immediately afterwards, which may result in a rapid-responder bias. However, rapid-responder bias may be an issue if the required sample is very small or specific, which is not the case in our study. In addition, Prolific used several strategies to reduce rapid-responder bias and to equally distribute study places among active users. Due to the fact that the ability to speak English is one of the inclusion criteria in our study, the generalizability of our findings may be limited to the US and other English-speaking countries. Therefore, similar interventions should be implemented in other countries in order to generalize our findings to other populations.

Moreover, as our study is a simulation of what occurs in the actual online environment, with individuals who are not necessarily representative of those who use social media platforms to debate vaccines, this research might potentially be transferred to the actual social media environment. As far as the social media platform allows, we could promote the SAS video and link a questionnaire to viewers of the video. In many instances, social media networks also provide demographic insights that might be utilized. Additionally, we could examine comments provided below the video.

## Ethics and dissemination

### Ethics and confidentiality

On 5 January 2022 the trial was registered with German Clinical Trials Register (www.drks.de) with the number DRKS00027938. On 4 May 2022, the Ethic Committee of the Heidelberg University Hospital approved this study (S-163/2022). Any alterations to the protocol will be communicated to the aforementioned Ethics Committee. The same committee is in charge of data monitoring and conducts periodic reviews of the protocol's progress and compliance with the declarations. The Declaration of Helsinki's principles and Good Clinical Practice guidelines will be followed.

Written informed consent will be obtained from all participants in this study. Before participants may begin the survey, they must read the study information and consent form on Prolific. The information and consent form details the study's objectives, as well as potential risks and benefits. Additionally, participants will be provided with a link to Prolific's data privacy policy, which they consented to while joining with Prolific. We will offer participants with the PI's (SB) and the Heidelberg Ethics Committee's contact information. We will inform participants that their names may be revealed to us if they email the PI. The study's scientists will maintain the confidentiality of this information. Consent will be obtained by checking a permission box on the consent form.

Only the study management will have access to the code of the pseudonymization. Data will be pseudonymized as soon as possible, according to § 35 Abs. 2 LDSG BW.

Names and all other personal and medical information are subject to confidentiality and to the regulations of the Federal Data Protection Act (Bundesdatenschutzgesetz – BDSG), the General Data Protection Regulation (Datenschutz-Grundverordnung – DSGVO) and State Data Protection Act (Landesdatenschutzgesetzes - LDSG). We will not collect any personal information (such as the participant's name or address) or medical information. Participants will be completely anonymous to the study investigators.

### Dissemination of research findings

The findings of the study will be published in peer-reviewed scientific publications and presented at scientific conferences.

## Ethics statement

The studies involving human participants were reviewed and approved by Heidelberg University Hospital's Ethics Committee, Medical Faculty, Heidelberg University (S-163/2022). The patients/participants provided their written informed consent to participate in this study.

## Author contributions

The pilot RCT protocol was designed and written by VH, CF, and SB. MA and TB assisted in the design of the participant identification plan and provided guidance on other critical study aspects, as well as in the intervention design. FB and SB are in charge of data collection, management, and statistical analysis. MA led the creation of SAS videos. Each author provided significant intellectual content to the written protocol and approved its final publication version. All authors contributed to the article and approved the submitted version.

## Funding

The study received no external funding. The study was funded by independent institutional resources available to Heidelberg Institute of Global Health.

## Conflict of interest

The authors declare that the research was conducted in the absence of any commercial or financial relationships that could be construed as a potential conflict of interest. The reviewer RMS declared a shared affiliation with the author FB to the handling editor at the time of review.

## Publisher's note

All claims expressed in this article are solely those of the authors and do not necessarily represent those of their affiliated organizations, or those of the publisher, the editors and the reviewers. Any product that may be evaluated in this article, or claim that may be made by its manufacturer, is not guaranteed or endorsed by the publisher.
